# HBMIRT: A SAS macro for estimating uni- and multidimensional 1- and 2-parameter item response models in small (and large!) samples

**DOI:** 10.3758/s13428-024-02366-8

**Published:** 2024-03-22

**Authors:** Wolfgang Wagner, Steffen Zitzmann, Martin Hecht

**Affiliations:** 1 Hector Research Institute of Education Sciences and Psychology, University of Tübingen, Europastraße 6, 72072 Tübingen, Germany; 2 Helmut Schmidt University Hamburg, Hamburg, Germany

**Keywords:** Bayesian IRT, Multidimensional IRT, Hierarchical priors, Small sample, PROC MCMC

## Abstract

Item response theory (IRT) has evolved as a standard psychometric approach in recent years, in particular for test construction based on dichotomous (i.e., true/false) items. Unfortunately, large samples are typically needed for item refinement in unidimensional models and even more so in the multidimensional case. However, Bayesian IRT approaches with hierarchical priors have recently been shown to be promising for estimating even complex models in small samples. Still, it may be challenging for applied researchers to set up such IRT models in general purpose or specialized statistical computer programs. Therefore, we developed a user-friendly tool – a SAS macro called HBMIRT – that allows to estimate uni- and multidimensional IRT models with dichotomous items. We explain the capabilities and features of the macro and demonstrate the particular advantages of the implemented hierarchical priors in rather small samples over weakly informative priors and traditional maximum likelihood estimation with the help of a simulation study. The macro can also be used with the online version of SAS OnDemand for Academics that is freely accessible for academic researchers.

## HBMIRT: A SAS macro for multidimensional item response models in small (and large!) samples

Psychometric assessment of categorical (in many cases dichotomous) test items is often based on item response theory (IRT), particularly in large scale studies such as the Programme for International Student Assessment (PISA; Organization for Economic Co-Operation and Development, 2016) or the Trends in International Mathematics and Science Study (TIMSS; Martin et al., 2020). IRT models can be considered as a special case of latent variable models with categorical indicators that load on one or more continuous latent variable(s) (Glockner-Rist & Hoijtink, 2003; Muthén, 2002). These models address a variety of different analysis goals regarding psychometric properties at the item- and test level such as the analysis of item discriminations, within- and between-item multidimensionality (Adams et al., 1997), and test reliability (person separation reliability; Andrich, 1982). In addition, the models can address questions regarding individual persons parameter estimates such as their precision (i.e., standard errors or posterior standard deviations).

Despite the great potential of IRT analyses, in many cases, they cannot be applied to empirical data due to insufficient sample size. In fact, for a unidimensional two parameter logistic (2PL) model (i.e., difficulty and discrimination are estimated for each item), samples sizes of *N* > 500 are recommended, and even larger sample sizes for more complex (e.g., multidimensional) models (Liu & Yang, 2018).

Besides model complexity, the specific estimation method has been shown to play a major role for the required sample size (Garnier-Villarreal et al., 2021). Bayesian methods have been shown to be advantageous over frequentist methods in small samples when prior distributions are specified in an advantageous way (Garnier-Villarreal et al., 2021; König et al., 2020). However, the specification of such a prior distribution can be challenging: A prior that is advantageous in one case may not be so in another case. Often, so called uninformative (or diffuse, flat, vague) priors are specified when no information is a priori available about the parameters to be estimated, which can lead to severe bias when samples are small (Smid et al., 2020; Zitzmann, Lüdtke, et al., 2021).

To mitigate this problem, the use of hierarchical prior distributions (also called adaptive informative priors) has specifically been suggested for IRT models when these models are used in small samples (Fujimoto & Neugebauer, 2020; Gilholm et al., 2021; König et al., 2020; Sheng, 2013). The idea behind this approach is that distributions for the parameters of a type of prior for model parameters (e.g., for a set of item discrimination parameters) are assumed. As a consequence, the prior distribution (e.g., for a specific item discrimination) is “adaptive” in such a way that it is adapted during the estimation process.

From this point of view, the question arises why such hierarchical Bayesian IRT models are not more routinely applied. One major reason for this might be that easy-to-use tools are not available in which users can specify such models without formulas as, for instance, in standard IRT tools (that do not allow for hierarchical Bayesian estimation) such as ConQuest (Wu et al., 2007), PROC IRT (SAS Institute Inc., 2018), the SPIRIT macro (DiTrapani et al., 2018) for SPSS (IBM Corp, 2019) or the many R packages (for an overview see Choi & Asilkalkan, 2019) such as the TAM package (Robitzsch et al., 2024) or the mirt package (Chalmers, 2012).

To provide users with such a tool, we developed a SAS macro based on PROC MCMC (SAS Institute Inc., 2018) that allows users to easily specify and estimate hierarchical Bayesian multidimensional IRT models (for user-specified IRT models with PROC MCMC see Ames & Samonte, 2015; Stone & Zhu, 2015): the HBMIRT^1^ macro for dichotomous items, which supports hierarchical priors for item intercept and item slope (i.e., item discrimination or factor loading) parameters in logistic (logit link) and normal ogive (probit link) models. We choose SAS PROC MCMC as it shows fewer memory limitations compared to different R packages and is computationally very efficient (Ames & Samonte, 2015) due to multithreading (which is however not available in the online version of SAS OnDemand for Academics). In the following, we explain the capabilities and features of the macro, including an automated procedure for monitoring convergence. Moreover, we compare the performance of the implemented hierarchical priors with different constant hyperparameters and maximum likelihood estimation in a small simulation study with unidimensional two-parameter logistic (2PL) models.

### The HBMIRT macro

In the following, we first give a short overview about the range of modeling options that are available within the HBMIRT macro. We then explain how different models can be specified with the HBMIRT macro with concrete examples with illustrated listings and outputs. We begin with the specification of a standard unidimensional 2PL model and increase the complexity of the model and the applied (hierarchical) priors step by step and explain why such model extensions can be useful. In the subsequent section, we introduce the use of the remaining macro options, for instance, regarding the convergence criteria, the naming of output data sets, and the possibility of specifying priors with constant hyperparameters instead of hierarchical priors.

### Modeling options

**Fig. 1:**
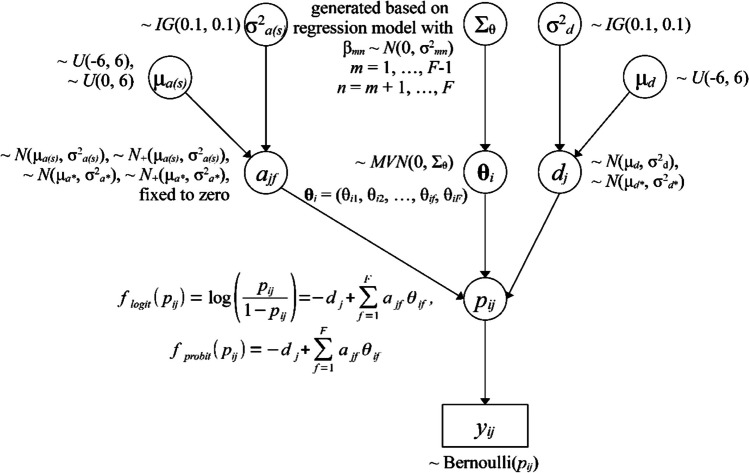
A directed acyclic graph of the general model and options

The HBMIRT macro allows users to specify a variety of uni- and multidimensional IRT models with binary outcomes. The general model and modeling options are shown in Fig. 1. It is essentially a multidimensional version of the 2PL model (M2PL; Reckase, 2009) – when the default logistic link function instead of the optional probit link function is used – with binary response *y*_*ij*_ for a person *i* on an item *j*. The responses *y*_*ij*_ are assumed to be a function of the person parameters θ_*if*_ for a person *i* on a factor (or dimension) *f* and the item parameters, namely the intercepts *d*_*j*_ and the discriminations or slopes *a*_*jf*_ (Chalmers, 2012). For both item intercept parameters and item slope parameters, normal prior distributions are specified. The parameters for at least one item per dimension are associated with a truncated normal prior *a*_*jf*_ ~ *N*_+_(µ_*a*_, σ^2^_*a*_), ensuring positive item slope parameters for identification purposes. The hyperparameters (µ_*a*_ and σ^2^_*a*_ or µ_*d*_ and σ^2^_*d*_, respectively) of the prior distributions for the item parameters can be defined as prespecified constants (µ_a*_, σ^2^_a*_, µ_d*_, σ^2^_d*_) or – in the case of hierarchical priors – as parameters with associated hyperprior distributions (µ_*a(s)*_, σ^2^_*a(s)*_, µ_*d*_, σ^2^_*d*_). If hierarchical priors are applied, the hyperparameters regarding the means of the prior distributions are uniformly distributed as µ_*d*_ ~ *U*(-6, 6 ) and µ_*a(s)*_ ~ *U*(-6, 6 ) or µ_*a(s)*_ ~ *U*(0, 6 ), respectively, and the hyperparameters regarding the variances are given inverse gamma distributions with shape and scale parameters both equal to 0.01: σ^2^_*a(s)*_ ~ *IG*(0.1, 0.1) and σ^2^_*d*_ ~ *IG*(0.1, 0.1) as default. Note that the hierarchical prior distributions including the hyperparameters can be changed by macro parameters. Further, in the case of the slope parameters, different sets (*S*) of hyperparameters with identical hierarchical prior distributions may be defined that are related to different sets of item slope parameters, for instance, a first set relating to slope parameters regarding the second dimension – *a*_*j*2_ ~ *N*(µ_*a*(1)_, σ^2^_*a*(1)_) – and a second set relating to slope parameters regarding the first dimension – *a*_*j*1_ ~ *N*(µ_*a*(2)_, σ^2^_*a*(2)_). It is also possible to use hierarchical priors only for a subset of the item slope and intercept parameters and constant hyperparameters for the remaining priors – for instance, *d*_*j*_ ~ *N*(µ_d*_, σ^2^_d*_) for *j* > 10. In the case of (multidimensional) 1-parameter models, where all item slopes regarding a given dimension are assumed to be equal (i.e., *a*_*jf*_ reduces to *a*_*f*_), constant hyperparameters a_*f*_ ~ *N*_+_(µ_a*_, σ^2^_a*_) are used for the priors of the slope parameter(s). In the case of multidimensional models positive definite correlation matrices for the person parameters θ_*if*_ including the option to constrain selected correlations to zero are generated based on a regression approach (for details see Appendix A).

### Specifying IRT models with the HBMIRT macro

**Fig. 2:**
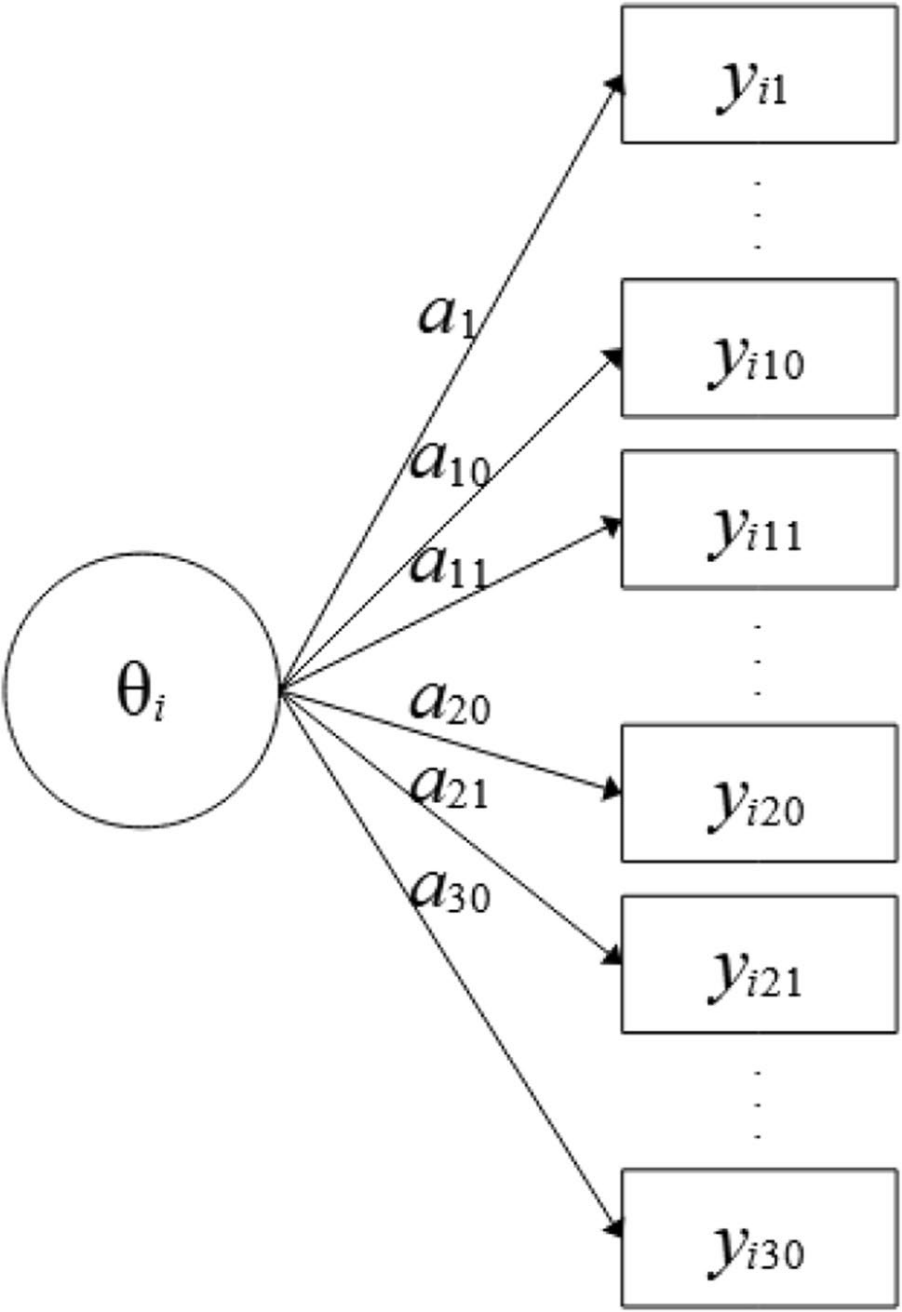
Unidimensional 2PL model with 30 indicators. Note. Item slope parameters are denoted as *a*_1_-*a*_30_. Item intercepts are not displayed.

Suppose we have assessed early numeracy (Dierendonck et al., 2021) with a standardized test in a sample consisting of 140 kindergarten children (the SAS listing to generate the data set and the listing for the analyses as well as the respective output are provided in Appendix B). The test encompasses three theoretical facets, each assessed by 10 items, namely counting, relations, and arithmetic. To illustrate the macro, we first estimate a unidimensional 2PL model (Fig. 2) with the hierarchical Bayesian approach (i.e., using hierarchical priors for the item parameters). Our data set *numeracy* in the SAS library *lib* comprises a unique numerical variable for each child (ID) named *idchild* and the variables *y1*-*y30* (counting *y1*-*y10*, relations *y11*-*y20*, and arithmetic *y21*-*y30*) containing the scored items (0 = false, 1 = correct).

**Unidimensional 2PL model.** To estimate our model, we use the following specification (Listing 1):

**Listing 1**

*“Standard” unidimensional 2PL model with hierarchical priors* 
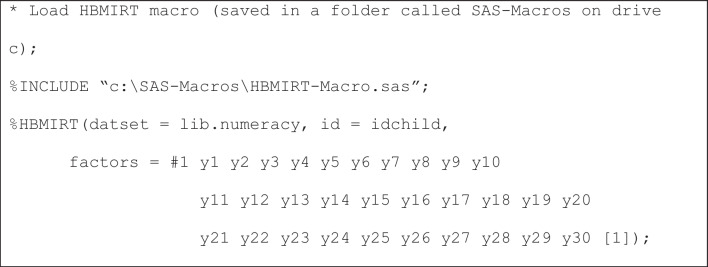


The 2PL model is the default choice. Therefore, what needs to be specified here are the name of the data set, the name of the ID variable, one factor (#1) with the respective items that load on that factor (*y*1-*y*30), and a label ([1]) that declares that the loadings (i.e., item slopes or *a*_*j*_ parameters with *j* = 1, …, 30) of the items *a*_*j*_ parameters share the same mean and variance hyperparameters. This way of modeling item slopes is consistent with the view that identical normal prior distributions would be specified in a non-hierarchical model. A similar assumption is made for the item intercepts (*d*_*j*_ parameters with *j* = 1, …, 30). It should be noted that the model is parameterized as *p*(*y*_*j*_ = 1) = logistic(-*d*_j_ + *a*_*j*_ · θ_*i*_), meaning that higher intercept values indicate lower probabilities for correct answers for an item with a positive slope and a given person ability.

**Fig. 3:**
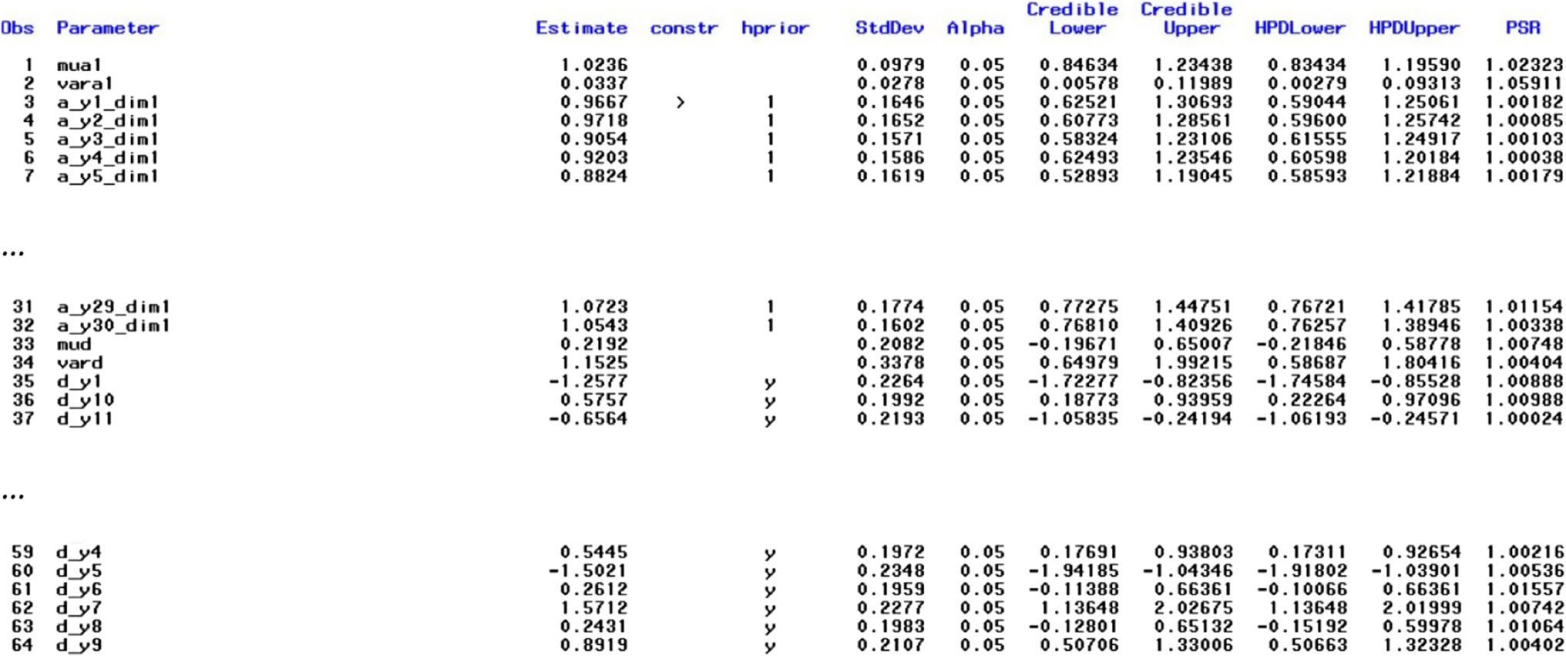
Output for unidimensional 2PL model presented in Listing 1 (the five right-most columns and lines 36-64 are excluded)

The results for this model are shown in Fig. 3. Each line – here denoted in SAS as *Obs* for “observation number” in the respective output data set – contains a parameter estimate. The name of the parameter can be found in the second column (Parameter) and the respective estimate and its posterior standard deviation in columns three (Estimate) and six (*StdDev*), respectively. The fourth column (*constr*) shows the constraints applied to identify the model or to avoid label switching (see also below). Only one positivity constraint was applied to the slope parameter of item *y*1 on dimension 1 (a_y1_dim1), which is the only assumed dimension here (therefore, all names of slope parameters have the suffix dim1). The slope parameters a_y1_dim1 … a_y30_dim1 correspond to those denoted as *a*_1_-*a*_30_ in Fig. 2, where “dim1” is denoted as θ. The fifth column (*hprior*) shows that hierarchical priors are used in the estimation. One set of hyperparameters (mean and variance) is used for all slope parameters and another set is used for all intercept parameters. The “y” in column *hprior* means that the item intercept (*d_y1*) belongs to the set of parameters with the same hierarchical prior (marked by “y” for “yes”, whereas unmarked intercepts would mean that they are not based on hierarchical priors), which are all intercept parameters, although they are not shown in Fig. 3. Columns eight and nine as well as columns 10 and 11 show credibility intervals (equal tail intervals and highest posterior density [HPD] intervals) at the specified alpha level (*alpha*, column seven, with default value .05). The right-most column in Fig. 3 (*PSR*) contains the potential scale reduction (PSR; Gelman & Rubin, 1992) for each parameter as described in Asparouhov and Muthén (2010) for the second half of the total chain (the first half of the chain is treated as a burn-in phase and always discarded for all calculations). If all PSR values fall below the cutoff (default PSR_conv = 1.1) then the column to the right of the PSR column (*converged*) – not shown in Fig. 3 – contains ones instead of zeros, indicating that the estimation has converged. If the expected sample sizes (ESS; Geyer, 1992; Kass et al., 1998) criterion is additionally used as a stop criterion (default ESS_conv = 0 means that ESS is not computed), convergence depends on both criteria. It is also possible to switch off the PSR criterion (PSR_conv=0) and to use only the ESS criterion. Note that a large ESS can improve the accuracy of the estimates (Zitzmann & Hecht, 2019; see also Zitzmann, Weirich, et al., 2021a, 2021b). Recently, a SAS macro called automcmc (Wagner et al., 2023) has been introduced that can be used to apply the identical stopping criteria as those in HBMIRT to IRT models that require user-defined PROC MCMC code and are not yet covered by HBMIRT.

In the IRT literature, item difficulties are often reported. It is also possible to obtain these parameters (*b* parameters), which can, due to the model parametrization *p*(*y*_*j*_ = 1) = logistic(-d_j_ + *a*_*j*_ · θ), be computed as *b*_*j*_ = *d*_*j*_ /*a*_*j*_ (Stone & Zhu, 2015) in unidimensional models (for multidimensional models see Reckase, 2009) using the output data set containing the generated posterior samples (called *outpost*). Convergence regarding these parameter estimates can be checked by applying the SAS ESS-autocall macro (which is also used in the HBMIRT macro if ESS is specified to monitor convergence)^2^.

*Different sets of prior distributions*. It might be reasonable to assume different hierarchical prior distributions (means, variances) for different item loadings; for example, depending on the specific facet these loadings belong to (counting y1-y10, relations y11-y20, and arithmetic y21-y30). In this case, the model is specified as follows (Listing 2):

**Listing 2**

*Unidimensional 2PL model with specific loading hierarchical priors for different sets of items* 
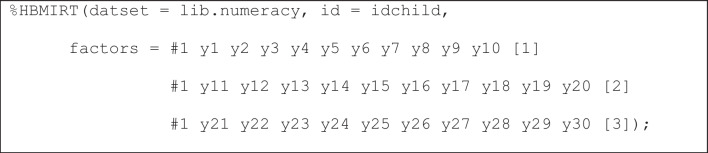


**Fig. 4:**
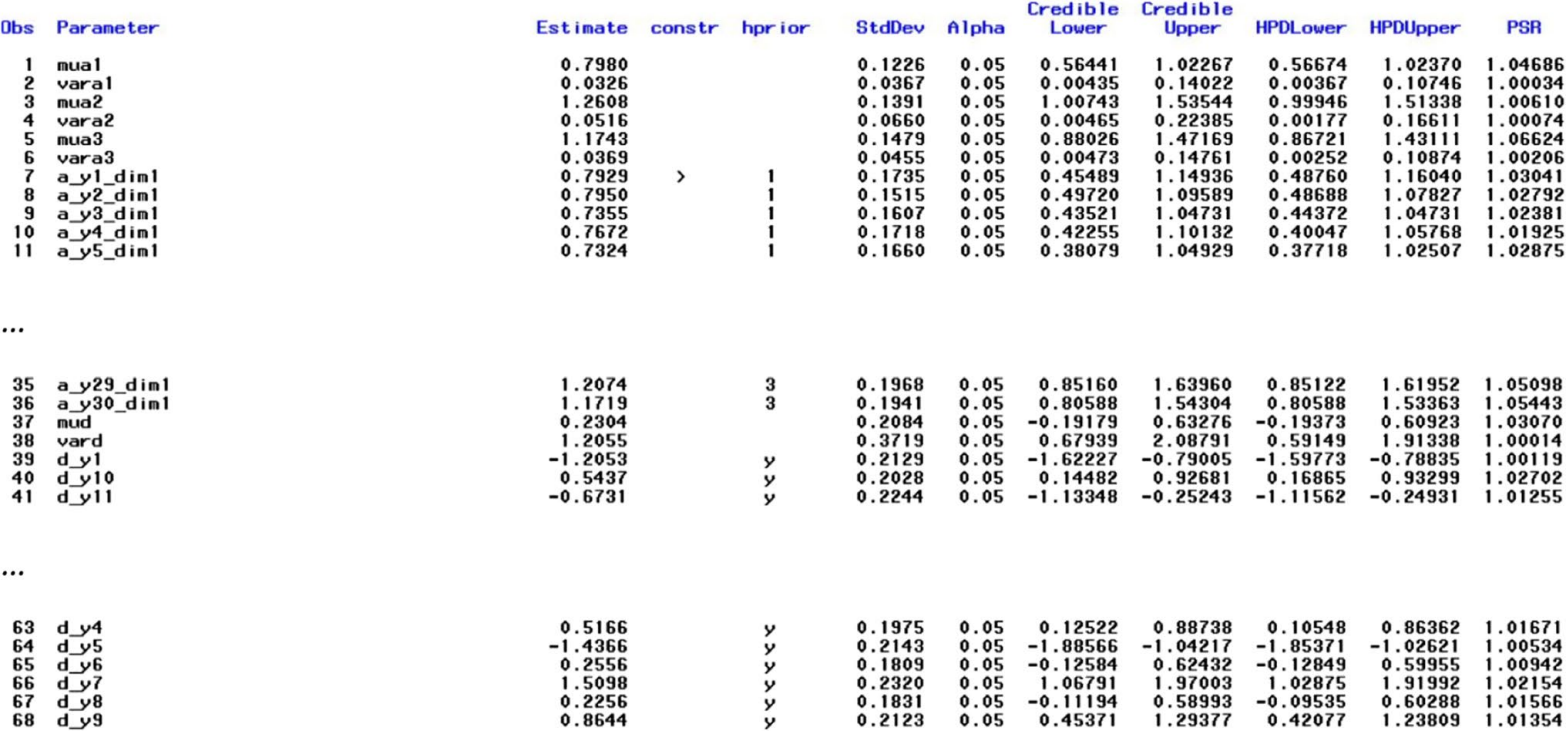
Output for unidimensional 2PL model presented in Listing 2 (the five right-most columns and lines 41-68 are excluded)

Here, three different means and three different variances (denoted as mua1-mua3 and vara1-vara3, respectively, in the output shown in Fig. 4 lines 1-6) are estimated for three different sets of prior distributions (as shown in column 5, lines 7-36). This means that the hierarchical prior distributions for the loadings of each facet (denoted as *a*_1_-*a*_10_, *a*_11_-*a*_20_ and *a*_21_-*a*_30_, respectively, in Fig. 2) are allowed to have different means and variances (e.g., highest loadings on average for facet 1). Notice that it is still a unidimensional model because all item sets load on the same dimension (#1 for dimension 1).

*Excluding items from estimation with hierarchical priors*. It is also possible to exclude single items from the hierarchical specification. If, for instance, three items (say *y10*, *y20*, and *y30*) have been newly developed and, therefore, may have less desirable psychometric properties than the established items, these can be estimated with an individual (nonhierarchical) normal prior for each of these items (e.g., by default, prior_mean_a = 0, prior_var_a = 4) as shown in Listing 3. In the output, the zeros in the *hprior* column would indicate that the respective item slopes do not fall under the umbrella of common hierarchical prior.

**Listing 3**

*Exclusion of items from estimation with hierarchical priors* 
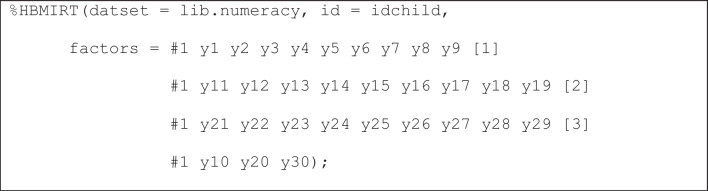


Regarding the intercepts, multiple sets of hierarchical priors are not allowed. However, as in the case of the loadings, single items can be excluded from the hierarchical prior by specifying the respective item names after the macro parameter duninf (e.g., duninf = y10 y20 y30). The intercepts of these items are then estimated using the specified (un)informative normal prior (e.g., with the default values prior_mean_d = 0, prior_var_d = 4).

*Hyperpriors*. The default priors proposed by Stone and Zhu (2015) for the hyperparameters in the hierarchical specification are uninformative with a uniform distribution ranging from -6 to 6 for the mean and an inverse gamma distribution with shape and scale parameters both equal to 0.01 for the variance. If the option muaconstr = yes is chosen, the mean is restricted to positive values. The priors for the hyperparameters can be modified by the macro parameters prior_mua and prior_mud for the means as well as prior_vara and prior_vard for the variances of the normal prior distributions of the item slope and intercept parameters. For instance, a more informative hyperprior (König et al., 2022) for the variance of the slope parameter prior could be specified as prior_vara = %STR(EXPON(ISCALE=0.4)) which would imply an exponential distribution with an inverse scale parameter equal to 0.4 or as a half-Cauchy distribution with location and scale parameters μ = 0 and σ = 2.5 by specifying prior_vara = %STR(CAUCHY(0, 2.5, lower=0)). Note that the %STR() function is necessary to mask the parentheses enclosing the distribution parameter(s) (here “iscale=0.4”) during macro compilation. If at least one of the options muaconstr = yes or the aconstr = yes (all slope parameters are constrained to be positive, see below) is used, the prior for the hyperparameter of the mean of the normal prior distributions for the item slope must exclude negative values. The default hyperprior here is specified as a uniform distribution ranging from 0 to 6 defined by the macro parameter prior_mua_ge0=%STR(UNIFORM(0, 6)).

*Estimation without hierarchical priors*. It is also possible to estimate a “traditional” Bayesian model with fixed prior means (as defined by prior_mean_a and prior_mean_d, respectively) and variances (as defined by prior_var_a and prior_var_d, respectively) for all *a* and *d* parameters by specifying the macro parameter priors = user instead of the default priors = hierarchical. It is important to note that the prior means and variances should be chosen with regard to the selected link function (link = logit or link = probit). The prior variance for *a* and *d* parameters with logit link refers to the logistic distribution with variance π^2^/3, whereas the probit link refers to the normal distribution with unit variance. So, for example, if mean and variance for *a* parameters with a logit link are chosen as prior_mean_a = 1 and prior_var_a = 4, the corresponding priors in a model with probit link should be approximately prior_mean_a = 1 / (π^2^/3)^1/2^ ≈ 0.55 and prior_var_a = 4 / (π^2^/3) ≈ 1.22.

*Model identification, label switching*. To identify the model, by default (aconstr = auto), one loading for each dimension is constrained to be positive. In some cases (e.g., if this loading is close to zero or the model is quite complex) label switching can appear because, for instance, in a unidimensional model, the likelihood for a set of loadings and the same set of loadings multiplied by -1 is identical. To avoid such problems, individual loadings can be constrained to be positive by adding the “>” sign after the item (without a blank). For example, “#1 y1> y2> y3> y4” means that y1-y3 are constrained to have positive slope parameters, whereas this is not the case for y4. Alternatively, all loadings can be constrained to be positive by setting aconstr = all. The positivity constraint is realized by truncating the specific normal prior distribution(s) at zero (see also Gilholm et al., 2021). This can also be applied to the hyperparameters for the means of the slope parameters by setting muaconstr = yes.

**Fig. 5:**
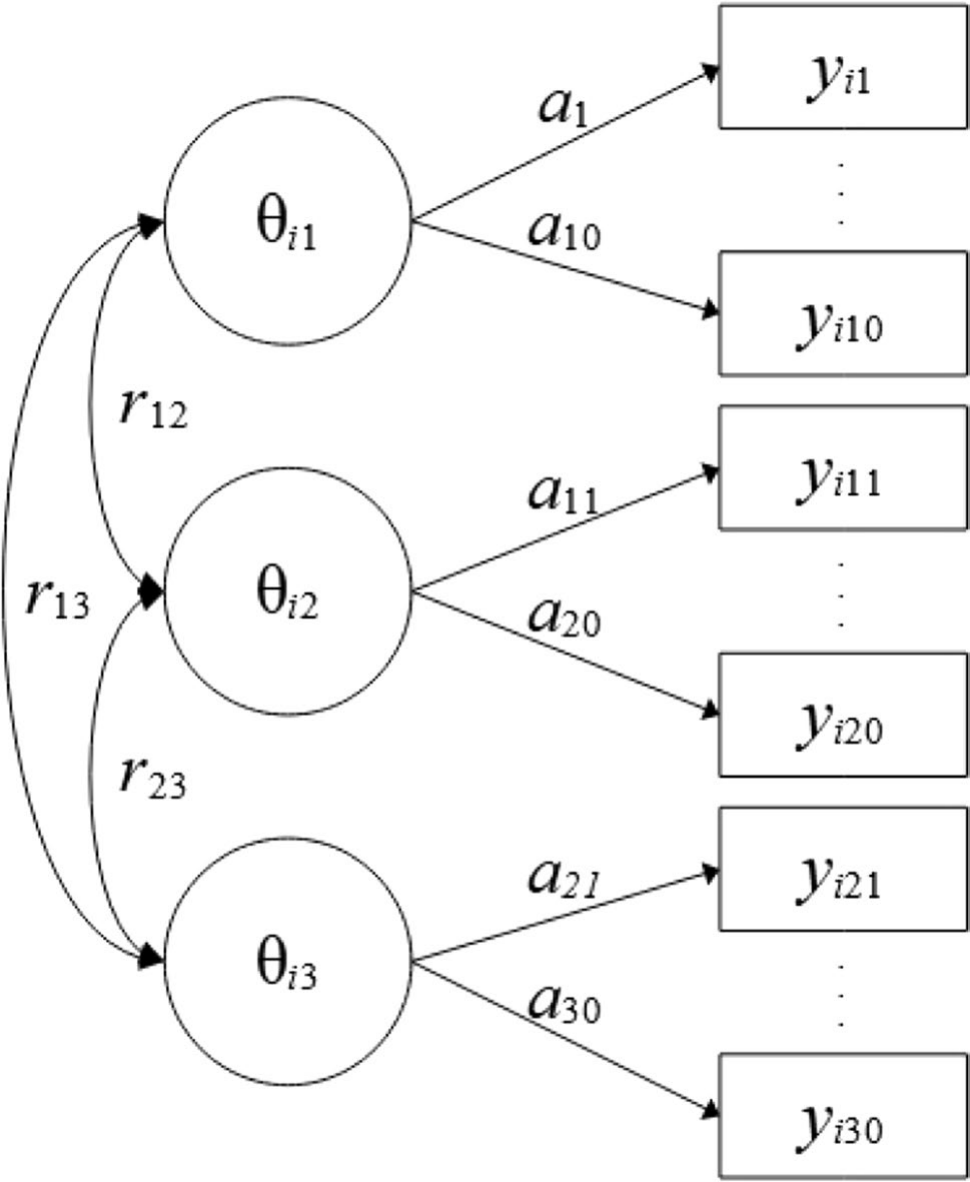
Three-dimensional 2PL model with 30 indicators. Note. Item slope parameters are denoted as a_1_-a_10_, a_11_-a_20_ and a_21_-a_30_ for dimensions 1 to 3 (denoted as θ_i1_- θ_i3_), respectively. Item intercepts are not displayed.

*Multidimensional models*. A multidimensional 2PL model with the three facets represented as different (correlated) dimensions (Fig. 5) can be estimated as follows (Listing 4):

**Listing 4**

*Three-dimensional model* 
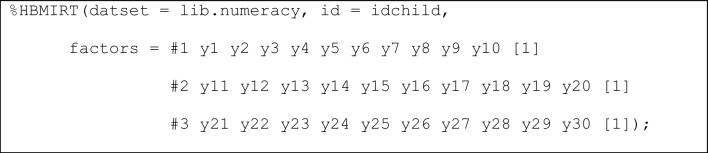


The factors are assumed to be intercorrelated by default (covar = free). If all correlations among factors should be constrained to zero, covar = diagonal can be specified. It is also possible to free correlations among factors, for example, by specifying covar = 1#2 1#3. Here, correlations between factors 1 and 2 (1#2) as well as between factors 1 and 3 (1#3) are estimated, whereas the correlation between factors 2 and 3 is fixed to zero (as 2#3 is not mentioned). If correlations among factors are estimated, the prior distributions of the correlations are given by default (rdist = yes). The prior distribution for the correlation matrix can be modified by choosing different values for var_beta (default: var_beta = 1) with smaller values implying distributions closer around zero.

In Listing 4, a global distribution of all item loadings is assumed. Factor-specific hierarchical prior distributions for the loadings can be specified by renaming [1] to [2] and [3] for the second and third factor (denoted as #2 and #3, respectively) in Listing 4. This would be in line with the population model used to generate the data (with loadings on factors 1-3 sampled from normal distributions with means 1, 1.5, and 2, respectively).

**Fig. 6:**
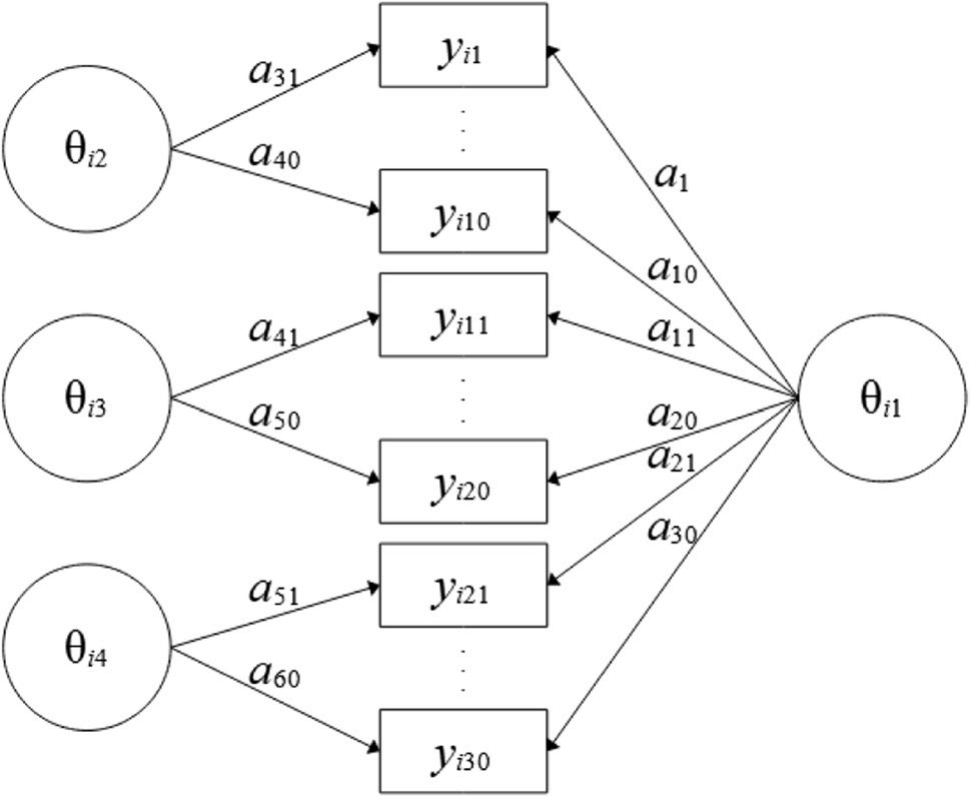
Four-dimensional M2PL bifactor model with 30 indicators. Note. Item slope parameters are denoted as a_1_-a_30_ for the general dimension (θ_i1_) and a_31_-a_40_, a_41_-a_50_ and a_51_-a_60_ for the specific dimensions 2 to 4 (denoted as θ_i2_-θ_i4_), respectively. Item intercepts are not displayed

Finally, a bifactor model (Fig. 6) with one general competence factor (with a specific hierarchical prior for the loadings) and three facet-specific factors (all factors are specified as uncorrelated by covar = diagonal) can be estimated. As each indicator is assumed to depend on two dimensions (i.e., loads on two factors) two slope parameters and one intercept parameter per indicator are estimated per indicator. Therefore, the probability of solving item 1, for instance, is modelled as *p*(*y*_1_ = 1) = logistic(-*d*_1_ + *a*_1_ · θ_*i*1_ + *a*_31_ · θ_*i*2_). Such models can be seen as multidimensional extensions of the 2PL (M2PL; Reckase, 2009) model. The specification of the respective bifactor model is shown in Listing 5.

**Listing 5**

*Four-dimensional bifactor model* 
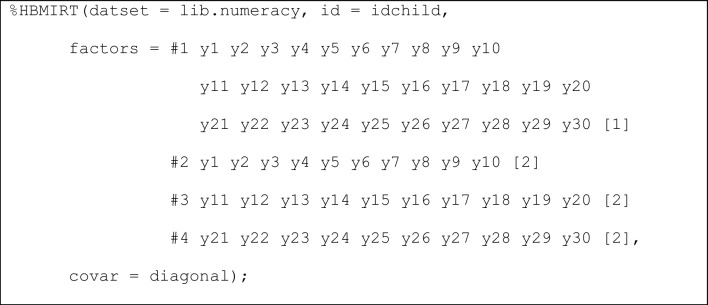


*Starting values*. Starting values are given by default as *a*_*j*_ = 1 for slope parameters (loadings) and *d*_*j*_ = 0 for intercept parameters and *r* = 0 for correlations among factors that are not fixed to zero. It is, however, possible to customize the starting values, which can be helpful when factors correlate. Reasonable starting values for correlations can be assigned in squared brackets directly after the respective factor pair to accelerate the estimation process, for instance, covar = 1#2[0.5] 1#3[0.4] (in this case, the svindat option – see below – is skipped). The correlation matrix implied by the starting values (including assumed uncorrelated factors) has to be positive definite. The respective regression weights used to generate the multivariate normal distribution for the person parameters (see above) are estimated with PROC CALIS based on the specified correlations. Starting values for all parameter estimates can also be given by SAS data sets mentioned under svindat or svlastitdat. The svlastitdat option is particularly helpful if more iterations are needed to increase the precision of estimates (smaller potential scale reduction [PSR] or larger effective sample size [ESS]). In this case, the data set from a prior model estimation specified under svlastitout (svlastitout = svlastitout by default) containing all relevant parameter estimates from the last iteration are used as starting values for a next chain of iterations. The resulting iterations can be combined by stacking both outpost files (e.g., outpost = outpost1 in first step, outpost = outpost2 in second step) to one data set (e.g., DATA outpost_combined; SET outpost1 outpost2;). It is also possible to specify user-defined starting values in a specific SAS data set by the svindat option (e.g., svindat = starting_values). The svindat data set has to contain starting values for all parameters to be estimated (except the person parameters). A simple way to generate a data set with all necessary variables is to estimate a given model with only a few iterations and use the resulting svoutdat data set (svoutdat = svoutdat as default) after updating the starting values as intended. Whenever models with hierarchical priors are estimated (and not all starting values are given in a data set), starting values are generated by first automatically estimating a model with constants (defined under prior_mean_a, prior_var_a, prior_mean_d, and prior_var_d) as parameters for the prior distributions. Because these are only starting values that thus should not have an impact on the final results, the use of rather informative priors is recommended, in particular in small samples where uninformative priors may otherwise lead to extreme parameter estimates in some cases. Applying different sets of starting values can also be used to check convergence based on several chains. For instance, a fixed number of (burn-in) iterations (nbi = 5E3, iterationsteps = 1E4; for details see below) could be specified without applying stopping criteria (PSR_conv = 0, ESS_conv = 0), so that based on each set of starting values HBMIRT would generate 10,000 draws from the given chain. Note that different names for the outpost data sets (e.g., outpost = chain1outpost, outpost = chain2outpost etc.) must be specified. The convergence across the different chains can then be checked with the SAS GELMAN-autocall macro.

*Output data sets*. Besides the above-mentioned starting values-related data sets, the HBMIRT macro generates output data sets for posterior summaries and credible intervals that are printed in the SAS output window by default (output = yes). Further, a data set containing the posterior distributions of all (hyper)parameter estimates except the person parameters is available (outpost = outpost). The latter are stored in a data set at an aggregate level (eap = eap) as expected a posteriori (EAP) estimates and their posterior standard deviations (i.e., the means and the standard deviations of the posterior distributions) computed from the second half of the total chain. The estimated EAP reliability for each dimension is included in the output data set. If ratioCPO is set to a value larger than zero, an additional data set (CPOdat = CPOdat) is generated which contains item and test level information in terms of the natural logarithms of the conditional predictive ordinate (CPO). The calculation is done as described by Stone and Zhu (2015). This information can be used for model comparisons using a pseudo Bayes factor approach (Geisser & Eddy, 1979). It should be noted that this approach is a very reliable model selection approach for IRT models with hierarchical priors (Fujimoto & Neugebauer, 2020) and even more reliable than the deviance information criterion (DIC; Spiegelhalter et al., 2002), which is generated by default as -2 · M(log likelihood) + 2 · Var(log likelihood) from the second half of the chain^3^. Further, in their simulation study, Fujimoto and Falk (2024) found that CPO-based Log-Predicted Marginal Likelihoods (LPMLs) showed an acceptable performance in IRT model selection regarding dimensionality (unidimensional versus multidimensional models including bifactor and two-tier structures) that was superior to the DIC and comparable to the Watanabe–Akaike Information Criterion (WAIC; Watanabe, 2013), although being inferior to the Pareto-Smoothed Importance Sampling-Leave-One-Out Cross-Validation (PSIS-LOO; Vehtari et al., 2022) that was however also the most computationally demanding to calculate.

*Missing values*. In empirical studies it is often the case that participants do not respond to every task or that several booklets were used with different subsets of the item pool (planned missingness). This means that the data set contains variables with missing values. As it may not be adequate to code missing values on items as incorrect (i.e., as zero) in the case of non-response (Pohl et al., 2014; Rose et al., 2016) – but see Robitzsch (2021) for arguments against treating non-response in terms of the latent ignorability assumption – and in the case of missingness by design, it is useful to rely on procedures that assume missingness at random (MAR; Rubin, 1976). This is the default in PROC MCMC (SAS Institute Inc., 2018), and the HBMIRT macro is designed to handle cases with this type of missingness.

### Further macro parameters to control estimation and customize output

In addition to the automatic monitoring of convergence (by specifying the PSR and/or ESS as the convergence criterion), the maximum number of iterations (maxnmc = 1E5 = 100,000 by default) after a number of burn-in iterations (nbi = 5E3 = 5,000 by default) prior to the first block of iterations (see iterationsteps option below) can be specified. Also, thinning (i.e., using only every n^th^ iteration) can be specified (thin = 1 by default, i.e., all iterations are used) in order to reduce the autocorrelation of the chain. However, thinning is only needed when there is not enough memory capacity to conduct the analysis (Link & Eaton, 2012). The check for convergence is applied after a specified number of iterations (iterationsteps = 5E3 = 5,000 by default). Regarding the sampling algorithm, we relied on the PROC MCMC default which is a random walk Metropolis with normal proposal for all parameters in our case except for the variance of the *d*-parameters (conjugate sampling) when a hyperprior is applied. Proposal tuning should generally be applied (maxtune = 100 loops – which is the maximum allowed number in PROC MCMC – for estimation with user-specified priors or hmaxtune = 100 for estimation with hierarchical priors by default), particularly when multidimensional models are estimated. The maximum number of tuning iterations can also be specified (ntu = 500 iterations – which is the maximum allowed number in PROC MCMC – for estimation with user-specified priors or hntu = 500 for estimation with hierarchical priors by default). As the tuning step implemented in PROC MCMC can be time consuming, the number of iterations for the estimation of the model parameters (iterationsteps) for a given PROC MCMC run should not be chosen too small, because each “iteration step” starts with another tuning step. When a model needs many steps, it might be reasonable to abort the job and choose a larger number of iterations for each step. Note that oftentimes SAS has to be closed to stop PROC MCMC (the “core” of the macro), because SAS does not respond to clicks on the break button. It is thus useful to start a new SAS instance for each job.

## A simulation study

We tested the performance of the method applied by the HBMIRT macro in a small simulation study with a unidimensional 2PL model with three different sample sizes (*N* = 50, *N* = 100, and *N* = 500) and a common number of items (*k* = 25). Item and person parameters were randomly drawn from normal distributions: *a* ~ *N*(1, 0.04), *d* ~ *N*(0, 1), and θ ~ *N*(0, 1) for each data replicate. The distribution of the slopes in our study closely approximates the log-normal distribution with μ = 0 and σ^2^ = 0.04 (mean and variance: E[*a*] = 1.02, Var[*a*] = 0.04) used in a simulation study based on a 2PL model by Monroe (2021). It is also comparable to the log-normal distribution with μ = 0 and σ^2^ = 0.06 in König et al. (2020) regarding the mean (E[*a*] = 1.03) which, however, shows higher variance (Var[*a*] = 0.07). Zhang and Zhao (2019) applied a uniform distribution ranging from 0.7 to 1.5 to generate discrimination parameters in their simulation study, which implied a (slightly) higher mean and variance (E[*a*] = 1.15, Var[*a*] = 0.05) compared to our study. Item difficulties were based on normal distributions in all three simulation studies with μ = 0 and σ^2^ = 1 (König et al., 2020; Zhang & Zhao, 2019) or σ^2^ = 0.56 (Monroe, 2021). The item difficulty distribution (*b* = -*d*/*a*) in our study based on uncorrelated item intercepts with *d* ~ *N*(0, 1) and slopes *a* ~ *N*(1, 0.04) has an identical mean (μ = 0) but a (slightly) larger variance (σ^2^ = 1.15).

The data sets were analyzed by a frequentist maximum likelihood approach (SAS PROC IRT with logistic link and option RESFUNC = nominal to get intercept estimates instead of difficulty estimates) and the HBMIRT macro with four different settings for the priors—uninformative: *a* ~ *N*(1, 100), *d* ~ *N*(0, 100); informative: *a* ~ *N*(1, 4), *d* ~ *N*(0, 4); hierarchical with hyperparameters with assigned hyperprior distributions (µ_a_ ~ *U*(-6, 6), σ^2^_a_ ~ *IG*(0.01, 0.01), µ_d_ ~ *U*(-6, 6), σ^2^_d_ ~ *IG*(0.01, 0.01) ; correct priors reflecting perfect prior knowledge of the population distributions: *a* ~ *N*(1, 0.04), *d* ~ *N*(0, 1)—and default settings for parameter constraints (one item with positivity constraint) and convergence (PSR < 1.1; ESSconv = 0, i.e., ESS criterion not applied). A total of 100 replicates were generated and then analyzed with each estimation approach and prior setting.

The results are presented in Table 1. With regard to the slope parameters, a rather large average bias (bias ≥ 0.215) was observed for very small samples (*N* = 50) except for the hierarchical Bayesian approach (bias = 0.050, though still statistically significantly larger than zero, 95% CI [0.019, 0.079]) and, as expected, for the approach with correct priors (both upper limits of the 95% CIs were below the lower limits of all other approaches in this condition). With *N* = 100, bias was negligible for the maximum likelihood (ML) approach and the Bayesian approaches with hierarchical and correct priors (bias ≤ 0.040 and overlapping 95% CIs) but large for the Bayesian approaches with uninformative and informative priors (bias ≥ 0.236; lower limits of the 95% CIs larger than the respective upper limits of all other approaches). In the large sample condition (*N* = 500), bias was negligible for all estimation approaches (bias ≤ 0.052; but statistically significantly different from zero for the estimation with (un-)informative priors). The estimated variances of the parameter estimates (squared *SE* for ML, and posterior variance for Bayesian approaches) and the root mean squared errors (RMSE) were extremely large, in particular for ML in the *N* = 50 condition. A closer look at the results showed that this was partly due to a few very extreme ML estimates: The 99^th^ percentile for the variances were 2.16 and 1.85 for the *a* and *d* parameters, respectively, which is far below the average variances of 29.41 and 57.88, respectively. Therefore, we additionally report the percentiles (5^th^, 50^th^, and 95^th^) for the length of the 95% confidence intervals (ML estimate ± 1.96 ∙ *SE*) and credibility intervals (highest posterior density, HPD, and equal tail intervals). In all conditions the upper limits for the variances for both *a* and *d* parameters of the 95% CIs for the hierarchical and correct prior specification were below the respective lower limits of all other approaches. This was also the case regarding the accuracy of the estimation (RMSE) with the exception of the *d* parameters in the *N* = 500 condition. Regarding the credibility intervals, the results showed acceptable coverage rates for the HPD intervals which – as they are always smaller than the equal tail intervals (Congdon, 2006) – were preferable over the equal tail intervals here. Therefore, we refer only to the HPD intervals in the following. Although the coverage rate was mostly acceptable across the conditions and the estimation approaches (with the exception of the Bayesian approach with uninformative priors and *N* = 50), we found large differences regarding the length of the intervals, which was on average markedly smaller for the hierarchical Bayesian approach compared with all other approaches with the exception of the correct prior specification and which is in line with the results for the variances. The same was true for the percentiles. This means that even in the case of *N* = 500, the hierarchical Bayesian approach outperformed ML estimation in this regard, with 3% compared to 31% larger average 95% CIs than those from the Bayesian approach with correct priors. Whereas in the *N* = 50 condition, the 95% CIs from the hierarchical Bayesian approach were 44% larger than those from the Bayesian approach with correct priors, in the *N* = 100 condition, they were only 18% larger than those from the Bayesian approach with correct priors.

**Table 1:** Results from simulation study for maximum likelihood (ML) estimation and Bayesian estimation with uninformative priors, informative priors, hierarchical priors, and correct priors

Sample size	Estimation	Bias	*M *(*SE*^2^, posterior variance)	RMSE	Coverage (95% CI)	Length CI			
						*M*	Percentile		
							5	50	95
Item slope (*a*)									
*N* = 50	ML	0.215 [0.142, 0.307]	29.408 [0.522, 78.021]	2.048 [0.904, 3.006]	.966	2.886	1.362	1.885	3.980
	uninf.	0.968 [0.903, 1.031]	1.394 [1.284, 1.512]	1.730 [1.608, 1.859]	.906 (.846)	3.727 (3.893)	2.065 (2.095)	3.059 (3.148)	7.448 (8.180)
	inform.	0.439 [0.406, 0.470]	0.436 [0.430, 0.442]	0.790 [0.768, 0.811]	.937 (.911)	2.458 (2.526)	1.704 (1.736)	2.359 (2.411)	3.543 (3.681)
	hier.	0.050 [0.019, 0.079]	0.072 [0.067, 0.077]	0.249 [0.233, 0.265]	.948 (.947)	1.018 (1.051)	0.777 (0.796)	0.978 (1.006)	1.380 (1.426)
	correct	0.001 [-0.005, 0.008]	0.034 [0.033, 0.034]	0.182 [0.177, 0.186]	.949 (.947)	0.709 (0.719)	0.664 (0.673)	0.707 (0.717)	0.756 (0.768)
*N* = 100	ML	0.041 [0.021, 0.062]	0.140 [0.135, 0.147]	0.381 [0.365, 0.398]	.959	1.407	1.004	1.313	2.079
	uninf.	0.321 [0.293, 0.349]	0.265 [0.234, 0.314]	0.651 [0.597, 0.717]	.931 (.907)	1.765 (1.816)	1.202 (1.218)	1.606 (1.641)	2.732 (2.842)
	inform.	0.236 [0.213, 0.259]	0.179 [0.176, 0.182]	0.486 [0.466, 0.505]	.940 (.923)	1.583 (1.620)	1.146 (1.163)	1.514 (1.548)	2.243 (2.308)
	hier.	0.011 [-0.009, 0.031]	0.042 [0.039, 0.045]	0.199 [0.189, 0.211]	.936 (.941)	0.781 (0.803)	0.578 (0.589)	0.774 (0.790)	1.024 (1.053)
	correct	0.000 [-0.007, 0.007]	0.029 [0.029, 0.029]	0.168 [0.163, 0.172]	.949 (.952)	0.660 (0.670)	0.615 (0.623)	0.660 (0.670)	0.707 (0.718)
*N* = 500	ML	0.008 [0.000, 0.016]	0.023 [0.023, 0.024]	0.154 [0.148, 0.160]	.946	0.593	0.475	0.581	0.753
	uninf.	0.052 [0.043, 0.061]	0.025 [0.025, 0.025]	0.171 [0.164, 0.177]	.930 (.935)	0.598 (0.612)	0.468 (0.479)	0.586 (0.599)	0.774 (0.795)
	inform.	0.049 [0.041, 0.058]	0.025 [0.025, 0.025]	0.168 [0.162, 0.174]	.932 (.934)	0.595 (0.609)	0.467 (0.477)	0.580 (0.593)	0.766 (0.784)
	hier.	0.004 [-0.005, 0.012]	0.015 [0.015, 0.015]	0.128 [0.124, 0.131]	.922 (.930)	0.462 (0.476)	0.373 (0.384)	0.457 (0.470)	0.568 (0.585)
	correct	0.002 [-0.003, 0.008]	0.014 [0.014, 0.014]	0.121 [0.117, 0.125]	.933 (.944)	0.450 (0.462)	0.392 (0.401)	0.447 (0.459)	0.516 (0.596)
Item intercept (*d*)									
*N* = 50	ML	0.058 [-0.048, 0.196]	57.881 [0.321, 170.217]	2.945 [0.605, 4.676]	.969	2.548	1.178	1.520	3.065
	uninf.	-0.014 [-0.058, 0.031]	0.436 [0.391, 0.493]	0.726 [0.661, 0.797]	.965 (.949)	2.134 (2.190)	1.257 (1.266)	1.766 (1.791)	4.244 (4.483)
	inform.	0.021 [-0.007, 0.050]	0.185 [0.181, 0.190]	0.410 [0.395, 0.425]	.957 (.956)	1.624 (1.652)	1.197 (1.213)	1.533 (1.559)	2.381 (2.446)
	hier.	-0.005 [-0.035, 0.026]	0.133 [0.129, 0.136]	0.348 [0.335, 0.363]	.951 (.956)	1.391 (1.420)	1.178 (1.200)	1.360 (1.390)	1.709 (1.739)
	correct	0.022 [-0.002, 0.046]	0.115 [0.114, 0.116]	0.333 [0.323, 0.343]	.952 (.954)	1.309 (1.327)	1.165 (1.176)	1.285 (1.302)	1.547 (1.563)
*N* = 100	ML	-0.010 [-0.033, 0.012]	0.099 [0.090, 0.111]	0.315 [0.293, 0.343]	.960	1.147	0.859	1.051	1.727
	uninf.	-0.008 [-0.034, 0.017]	0.123 [0.109, 0.143]	0.382 [0.334, 0.444]	.954 (.947)	1.219 (1.244)	0.868 (0.879)	1.100 (1.115)	1.901 (1.950)
	inform.	0.003 [-0.016, 0.023]	0.089 [0.087, 0.092]	0.288 [0.277, 0.298]	.952 (.952)	1.125 (1.144)	0.854 (0.864)	1.064 (1.081)	1.613 (1.649)
	hier.	-0.003 [-0.023, 0.018]	0.068 [0.067, 0.069]	0.257 [0.248, 0.266]	.941 (.946)	0.995 (1.016)	0.854 (0.870)	0.967 (0.987)	1.221 (1.248)
	correct	0.013 [-0.004, 0.030]	0.063 [0.062, 0.064]	0.249 [0.241, 0.257]	.946 (.951)	0.966 (0.980)	0.851 (0.862)	0.943 (0.957)	1.173 (1.189)
*N* = 500	ML	-0.002 [-0.012, 0.008]	0.016 [0.016, 0.017]	0.126 [0.121, 0.130]	.952	0.490	0.396	0.461	0.673
	uninf.	-0.004 [-0.014, 0.007]	0.016 [0.016, 0.017]	0.130 [0.124, 0.135]	.942 (.942)	0.478 (0.489)	0.375 (0.383)	0.452 (0.462)	0.664 (0.678)
	inform.	-0.004 [-0.015, 0.006]	0.016 [0.016, 0.017]	0.127 [0.122, 0.131]	.945 (.946)	0.476 (0.487)	0.373 (0.379)	0.452 (0.462)	0.654 (0.675)
	hier.	0.000 [-0.011, 0.010]	0.015 [0.014, 0.015]	0.122 [0.117, 0.127]	.940 (.944)	0.455 (0.468)	0.367 (0.377)	0.439 (0.453)	0.589 (0.608)
	correct	-0.001 [-0.011, 0.009]	0.014 [0.014, 0.014]	0.121 [0.117, 0.126]	.937 (.944)	0.449 (0.461)	0.372 (0.381)	0.434 (0.445)	0.582 (0.596)

In the conditions with samples sizes *N* = 100 and *N* = 500 all replications converged. In the condition with *N* = 50 one replicate was not estimable because of one item without variability. All other replications in this condition converged with Bayesian estimation and 94 replications converged with ML estimation. Coverage refers to 95% confidence intervals for ML estimation (ML estimate ± 1.96 · *SE*) and highest posterior density (HPD) or equal tail (reported in brackets) 95% credible intervals for Bayesian estimation. For bias, variance and RMSE 95% confidence limits are given in brackets based on 10,000 bootstrap samples of the average bias and squared error for each replicate (sampling with replacement across all replicates). Bias estimates with confidence limits that did not cover zero are printed in bold

Regarding the item intercepts, the results were overall very similar to those for the item slopes with two exceptions: (1) Only minor bias was observed across all conditions. (2) Although the hierarchical Bayesian approach showed the highest precision in terms of the smallest average length of the 95% CIs compared with the other estimation approaches (except the Bayesian approach with correct priors), this advantage was less pronounced than for the item slopes. Compared to the correct prior specification, the 95% CIs for the hierarchical Bayesian approach were only 6% larger on average across all sample sizes, whereas for ML estimation, this difference was 151%.

It should be noted that in Table 1 the term bias refers to average bias across replications for all items. Another important aspect is the dependence of bias on the size of the population parameter. It is a well-known fact that in Bayesian estimation with informative priors estimates are “pulled” towards the mean of the prior distribution (e.g., van de Schoot et al., 2017). In the case of a hierarchical Bayesian approach this effect is also expected as the variance hyperparameters of the priors will typically be rather small and thus quite informative. Therefore, we also investigated the linear association of bias and population parameters. The results for the bias of the item slope parameters are shown in Table 2. Regarding the association of bias and population parameters the results show statistically significant positive (ML—except for the *N* = 50 condition—, uninformative, informative prior) or negative (hierarchical and correct priors) linear associations for the *N* = 50 and the *N* = 100 condition. However, almost no statistically significant associations but for hierarchical and the correct priors were found in the *N* = 500 condition. The negative associations for the hierarchical and correct priors are in line with the assumption of a “shrinkage to the mean” of the prior distribution: In the *N* = 100 condition, for instance, applying the correct prior would be expected to lead to the bias (*â*_j_ – *a*_j_) = 0.000 + (-0.726) · (*a*_j_ – M(*a*_j_)) (the average bias was <0.001 as can be seen in Table 1). This means that the expected upward bias for a population parameter one unit below the average would be 0.726, or, equivalently, the expected downward bias for a population parameter one unit above the average would be -0.726.

**Table 2:** Linear associations of item slope bias and the size of the population parameter by sample size and estimation approach

	Model	Est.	*SE*	*p*	95% CI		*R*^2^
					lower	upper	
*N* = 50	ML	0.156	0.256	.544	-0.346	0.657	.000
	uninf.	1.093	0.129	<.001	0.841	1.345	.023
	inform.	0.297	0.061	<.001	0.177	0.417	.008
	hier.	-0.695	0.022	<.001	-0.738	-0.652	.317
	correct	-0.842	0.007	<.001	-0.856	-0.827	.840
*N* = 100	ML	0.084	0.036	.023	0.013	0.155	.002
	uninf.	0.428	0.050	<.001	0.329	0.526	.022
	inform.	0.243	0.041	<.001	0.163	0.323	.013
	hier.	-0.640	0.025	<.001	-0.689	-0.591	.406
	correct	-0.726	0.009	<.001	-0.743	-0.708	.731
*N* = 500	ML	-0.015	0.016	.341	-0.047	0.016	.000
	uninf.	0.030	0.017	.081	-0.003	0.063	.001
	inform.	0.024	0.017	.153	-0.009	0.057	.001
	hier.	-0.357	0.018	<.001	-0.392	-0.321	.305
	correct	-0.369	0.011	<.001	-0.390	-0.348	.362

Statistically significant (*p* < .05) estimates are printed in bold. The regressions were based on cluster-robust regressions (PROC SURVEYREG) with replications as clusters

With regard to the item intercepts (Table 3), the association between population parameter and bias was statistically significant for all approaches and all sample sizes with the same pattern as in the case of the item slopes (negative associations for the hierarchical and the correct priors, positive associations for all other approaches).

**Table 3:** Linear associations of item intercept bias and the size of the population parameter by sample size and estimation approach

					95% CI		
	Model	Est.	*SE*	*p*	lower	upper	*R*^2^
*N* = 50	ML	0.381	0.168	.026	0.051	0.711	.017
	uninf.	0.275	0.020	<.001	0.235	0.314	.147
	inform.	0.061	0.008	<.001	0.046	0.077	.023
	hier.	-0.081	0.008	<.001	-0.097	-0.065	.055
	correct	-0.111	0.005	<.001	-0.122	-0.100	.115
*N* = 100	ML	0.051	0.008	<.001	0.034	0.067	.027
	uninf.	0.107	0.011	<.001	0.085	0.129	.081
	inform.	0.041	0.006	<.001	0.028	0.053	.021
	hier.	-0.045	0.006	<.001	-0.056	-0.033	.031
	correct	-0.062	0.005	<.001	-0.072	-0.053	.065
*N* = 500	ML	0.006	0.003	.050	0.000	0.012	.002
	uninf.	0.015	0.003	<.001	0.009	0.022	.015
	inform.	0.008	0.003	.006	0.003	0.014	.005
	hier.	-0.012	0.003	<.001	-0.017	-0.006	.010
	correct	-0.016	0.003	<.001	-0.021	-0.011	.018

All estimates were statistically significant (*p* < .05). The regressions were based on cluster-robust regressions (PROC SURVEYREG) with replications as clusters

We also checked the coverage and (estimated) reliability of the EAP person parameter estimates from the Bayesian models (Table 4). As a reliability estimate (also computed by the HBMIRT macro by default), we used the variance of the EAP scores, which underestimates the true variance of the person parameters by (1 – reliability) percent (Mislevy et al., 1992). The results show an excellent coverage for the CIs^4^ based on estimate ± 1.96 · posterior *SD* from the Bayesian approaches with hierarchical and correct priors across all conditions, whereas for the Bayesian approaches with uninformative and informative priors, this was the case only in the *N* = 500 condition. A similar picture was found for the estimated reliabilities that were strongly underestimated (compared to the true reliabilities) when uninformative and informative priors were used, except for the *N* = 500 condition.

**Table 4:** Coverage and reliabilities (estimated and true) of the expected a posteriori (EAP) estimates from the Bayesian estimation

	Estimation	*N* = 50	*N* = 100	*N* = 500
Coverage (95 %)				
	uninformative	0.767	0.879	0.935
	informative	0.855	0.897	0.936
	hierarchical	0.948	0.948	0.946
	correct prior	0.950	0.950	0.947
Reliability (estimated)				
	uninformative	0.433	0.613	0.775
	informative	0.563	0.644	0.776
	hierarchical	0.770	0.787	0.809
	correct prior	0.810	0.800	0.811
Reliability (true)				
	uninformative	0.772	0.794	0.813
	informative	0.789	0.797	0.813
	hierarchical	0.811	0.808	0.814
	correct prior	0.811	0.810	0.815

Coverage refers to the relative amount of person parameters covered by the credible intervals defined as estimate ± 1.96 · posterior *SD*. Estimated reliability was based on the variance of the EAP person parameter estimates. True reliability was computed as the squared correlation between the person parameters and the respective EAP estimates

The simulation study was conducted using SAS 9.4 TS Level 1M6 on a Windows Version 1.0.19041 X64_10PRO platform notebook with an Intel Core i7-4600U CPU and 16 GB RAM. The average computation times for the estimation of the Bayesian models in the simulation study with the HBMIRT macro (that makes use of the multithreading capability of PROC MCMC) are given in Table 5. Further improvements regarding estimation time might be reached by specifying more appropriate starting values that should lead to faster convergence (i.e., less iterations on average). A very important aspect with regard to the computation time is a sufficient number of tuning loops for the proposal distributions (see, e.g., Hecht et al., 2021, who reported mixed results about the impact of the number of warmup iterations on the MCMC efficiency). On the one hand, a large number should lead to higher efficiency of the chain and, therefore, to a lower number of iterations needed to achieve a given ESS, for instance. On the other hand, the proposal tuning process might need a lot of computational resources. We are hesitant to give a general advice regarding the optimal settings for the proposal tuning. Based on a few experiments we skipped the tuning phase for non-hierarchical models (maxtune = 0) in our simulation study, but used proposal tuning for the hierarchical approach (hmaxtune = 100). If proposal tuning is needed, the number of iterations before the next check of the stopping criteria should not be chosen too small (e.g., iterationsteps = 2500), because the proposal tuning process starts again after each step. But if the proposal tuning phase can be skipped, (much) shorter “iteration steps” (e.g., iterationsteps = 500) can be chosen that my lead to a smaller number of total iterations given the stopping criteria. For instance, the first 10 replications of the model with the correct prior specification in our simulation study in the *N* = 500 condition with the applied setting (iterationsteps = 2500) all fulfilled the stopping criterion (PSRconv = 1.1) after the first “step” (average computation time: 3 min 3 s). With a smaller number of “iteration steps” (iterationsteps = 500), on average, the stopping criterion was fulfilled after 1,200 iterations (average computation time: 2 min 9 s). Another suggestion for reducing computational time in Bayesian estimation based on model reformulations was proposed by Hecht et al. (2020; see also Hecht & Zitzmann, 2020) and Merkle et al. (2021).

**Table 5:** Average computation time by model prior specification and sample size

	Model (prior specification)			
	uninformative	informative	hierarchical	correct
*N* = 50	3 min 24 s	1 min 46 s	9 min 53 s	1 min 17 s
*N* = 100	2 min 16 s	2 min 03 s	9 min 37 s	2 min 32 s
*N* = 500	2 min 49 s	2 min 43 s	14 min 60 s	3 min 11 s

## Discussion

During the last decades IRT has become the standard psychometric approach for scaling tests, in particular when dichotomously coded items (responses coded as correct or false) are used. The estimation of such models, however, typically requires rather large sample sizes which makes it difficult for applied researchers in many fields to make use of IRT. This drawback can be overcome by Bayesian IRT approaches with hierarchical priors that allow to estimate (multidimensional) IRT models in rather small samples as recent studies have demonstrated (Fujimoto & Neugebauer, 2020; Gilholm et al., 2021; König et al., 2020; Sheng, 2013). To date, such approaches might be under-used by applied researchers for two reasons: First, researchers may not be aware of the advantages of the Bayesian IRT framework with hierarchical priors. Second, it might be quite difficult for non-experts to set up such IRT models in general purpose (e.g. SAS; SAS Institute Inc., 2018) or specialized (e.g. Stan; Carpenter et al., 2017) statistical computer programs. For these reasons, we developed the HBMIRT macro for SAS with the central aim of user-friendliness. We tried to keep the model specification as simple as possible (comparable to other existing IRT tools such as SAS PROC IRT)—for instance, by adding many default settings—but still allowing for setting up quite complex multidimensional models. Further, we added an automated convergence control by applying a stop criterion analog to that in Mplus as default (which can easily be “switched off” or modified by the user). Finally, we gauged the macro performance (convergence, parameter recovery) in a simulation study.

To evaluate the method employed by the HBMIRT macro, we simulated data according to unidimensional 2PL models each with 25 items and different sample sizes (*N* = 50, *N* = 100, and *N* = 500). For each condition 100 data sets were generated and item parameters as well as person parameters were estimated based on Bayesian IRT models with the HBMIRT macro (uninformative, informative, hierarchical, and correct priors—the latter exactly matched the distributions from which the item parameters were drawn) as well as an ML-IRT model (SAS PROC IRT). The results showed that the convergence criterion was reached in virtually all cases and that the coverage rates for the item parameters were mostly acceptable for all estimation approaches (i.e., the 95% CIs included the respective population parameters in approximately 95% of the cases). Slope parameter estimates from the Bayesian approaches with (un-)informative priors were biased, particularly in the *N* = 50 condition, whereas estimates based on hierarchical and correct priors were (largely) unbiased across all conditions. Moreover, regarding the precision of the slope parameter estimates in terms of the length of the 95% CIs, the results showed a clear advantage of the hierarchical approach over all other approaches—except, of course, the correct prior specification. Interestingly, this was even the case in the *N* = 500 condition, where the hierarchical Bayesian approach outperformed ML estimation with 3% compared to 31% larger average 95% CIs for the slope parameter estimates than those from the Bayesian approach with correct priors. This means that even in this condition, the prior still had a non-negligible impact on the posterior distribution (see Table 2) of the slope parameter estimates and a higher accuracy in terms of the RMSE for the hierarchical Bayesian compared to all other approaches—except the correct prior specification—by “borrowing” information from other slope parameter estimates in the model. The results for the item intercepts showed a comparable but less pronounced pattern as in the case of the slope parameters for the different estimation approaches with the exception that bias was generally very small. Further, with regard to the EAP person parameter estimates from the Bayesian approaches, we found almost perfect coverages (95% CIs) and unbiased reliability estimates only for the estimation based on hierarchical priors and the correct priors across all conditions. The reliability estimates may be biased in both uninformative and informative prior approaches when these estimates are compared to their true values (determined by the squared correlations of EAP estimates with population parameters). This could result from a positive bias in slope parameters, which is “compensated” by a reduced variance of the EAP estimates, our chosen reliability index. Taken together, the main findings of our simulation study regarding the hierarchical Bayesian approach are in line with those from prior studies, which also found unbiased item parameter estimates even in small(er) samples (Fujimoto & Neugebauer, 2020; Sheng, 2013) and, additionally, highly accurate person parameter estimates (König et al., 2020, 2022) for hierarchical Bayesian IRT approaches.

## Conclusion

In particular with small and medium sample sizes and/or more complex models, less biased and more accurate parameter estimates in terms of a smaller RMSE can be expected with the Bayesian IRT approach with hierarchical priors compared to (un-)informative priors or ML estimation. With the HBMIRT macro for SAS the benefits of this approach are no longer restricted to experts with a strong background in Bayesian modelling. Uni- and multidimensional Bayesian IRT models with hierarchical priors for dichotomous items can easily be specified and estimated with automatic control of convergence by a default stop criterion. For more advanced users, the macro defaults can be modified, for instance, to estimate models with probit-link instead of the default logit-link, to alter the stop criterion (e.g., ESS as an alternative or additional criterion) or the hierarchical prior distributions, and to apply positivity constraints to specific item slope parameters or zero constraints on specific intercorrelations of latent dimensions (factors).

## Figures and Tables

**Figure 7:**
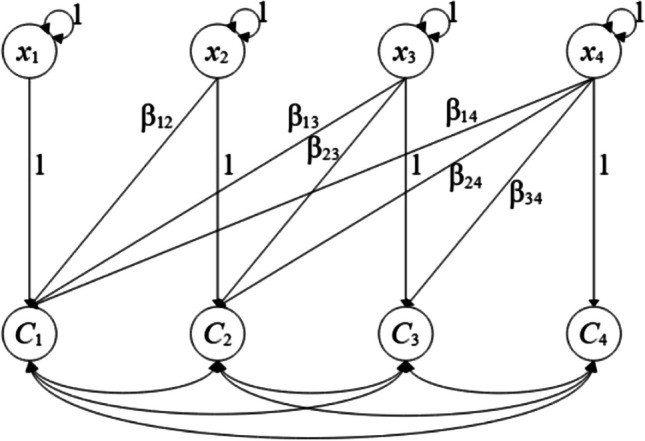
Model with latent composite scores (C_1_-C_4_) and component variables (x_1_-x_4_) for the generation of factor intercorrelations (example with four dimensions)

## Appendix A

### Generation of positive definite (constrained) correlation matrices

The generation of positive definite correlation matrices for the person parameters θ_*if*_ including the option to constrain selected correlations to zero is based on sampling (and constraining) regression coefficients assuming that a set of latent composite scores (e.g. *C*_1_– *C*_4_ in Fig. 7) is a weighted aggregate of an uncorrelated set of component variables with unit variance (*F* = 4 dimensions: *x*_1_– *x*_4_ in Fig. 7). The latent composite *C*_*f*_ is a weighted aggregate of *x*_*f*_, *x*_*f*+1_, …, *x*_*F*_. The regression weights are fixed to 1 for all β_*ff*_ and generated by normal distributions with zero means for the remaining weights. The variance var(β_*F*-1, *F*_) of the normal prior for the regression weight β_*F*-1, *F*_ of the last component variable (*x*_*F*_) on the second last composite score (*C*_*F*-1_) is fixed to 1 by default. Therefore, the expected average variance of *C*_*F*-1_ (across several draws) is given by var(*C*_*F*-1_) = 1 + var(β_*F*-1, *F*_) = 2 (the variance of the product of two normally distributed variables with mean zero, here the component variable and the regression weight, is equal to the product of both variances, see Goodman, 1960), whereas the expected average variance of the last composite score, *C*_*F*_, equals 1. To adjust the variance of the normal priors for the regression weights β_*mn*_ for *m* < *F*-1, *n* = *m* + 2, *m* + 3, …, *F* to the larger expected average variances of the composite scores with more “predictors”, var(β_*F*-1, *F*_) is multiplied by the ratio of var(*C*_*m*+1_) and var(*C*_*n*_). In the example with four factors in Fig. 7 the variance of the prior for β_24_ is automatically fixed to var(β_24_) = var(β_*F*-1, *F*_) · var(*C*_*m*+1_)/var(*C*_*n*_) = var(β_34_) · var(*C*_3_)/var(*C*_4_) = 1 · 2/1 = 2 – assuming that θ_*i*3_ and θ_*i*4_ are not specified as uncorrelated (otherwise the var(θ_3_) = 1 as β_34_ is fixed to zero). Correlations fixed to zero are realized by a constraint on the respective regression weight between the pair of composite scores to a value that neutralizes all impacts from other “predictors”. For instance, the covariance of *C*_2_ and *C*_3_ is given by cov(*C*_2_, *C*_3_) = var(β_24_) · var(β_34_) + var(β_23_) · 1. If this covariance (and the corresponding correlation) is fixed to zero, the respective regression coefficient is defined as β_23_ = -1 · β_24_ · β_34_ with var(β_23_) = var(β_24_) · var(β_34_). Finally, the correlations between the composite scores are computed as *r*_*mn*_ = cov(*C*_*m*_, *C*_*n*_)/(var(*C*_*m*_) · var(*C*_*n*_))^0.5^ and used to generate a multidimensional normal distribution with zero means and unit variances for the person parameters **θ**_*i*_.

## Appendix B

### Analysis Example (SAS code and output)

The following SAS job was used to generate the data for the illustrative example and store the data set in the folder “c:\HBMIRT_example”:

The following SAS job was used to analyze the data of the illustrative example (assuming that the data set was stored in the folder “c:\HBMIRT_example” and the macro HBMIRT-Macro.sas in the folder “c:\SAS-Macros\”):

The resulting outputs are presented in the following. Please note that information related to the estimated model – convergence status (1 = converged), time needed for estimation, EAP reliability, and DIC – instead of single parameters are repeated across lines. Lines are denoted as “Obs” in the output of the results data set (default: postsumint=postsumint) based on PROC PRINT.

Output for Listing 1

Output for Listing 2

Output for Listing 3

Output for Listing 4, prior distributions for correlations

Output for Listing 4, parameter estimates

Output for Listing 4b, parameter estimates

Output for Listing 5

## Footnotes

1 The %HBMIRT macro can be downloaded at: https://doi.org/10.17605/OSF.IO/D9YBU, accessed on 6 November 2023.

2 Note that the convergence and also the parameter estimates and credibility intervals from the HBMIRT macro are based on the second half of the chain, so the first half of generated samples in the outpost data set should be skipped in order to compare the results with those reported in the HBMIRT macro output. Advanced users may discard less than half of the samples after critical inspection of the chain to improve the estimation efficiency.

3 As the calculation of CPO is computational intensive and only values from the second half of the chain are used, it is reasonable to estimate a model in a first step, and then, use the resulting estimates from the last iteration (e.g. svlastitout = svlastitout1) as an input data set for a subsequent estimation with these estimates as starting values (svlastitdat = svlastitout1) and select, for instance, 10% (ratioCPO = 0.1) of the following second half of the outpost sample (the first half is always discarded for analyses – though available in the specified outpost data set) to compute the CPO.

4 For the person parameters, only the means and standard deviations are exported by the HBMIRT macro to avoid memory limitations in cases with larger sample sizes (e.g. 1,000 persons). Here, smaller numbers for the iterationsteps option can be chosen (e.g., iterationsteps = 1E3), which means that for each “step”, the posterior distribution for all person parameters contains 1,000 · 1,000 = 10^6^ estimates. After each step, for each person, the means and variances of the respective estimates are calculated for the two chain parts (first and second half of the chain) before the posterior distribution of the person parameters is deleted to save memory. Therefore, it is not possible to compute equal tail or HPD intervals for the person parameters based on the output of the HBMIRT macro.

## Declarations

### Open practice statement

The SAS code used to generate and analyze the data for the simulation study are included in Appendix C.

## References

[CR1] Adams RJ, Wilson M, Wu M (1997). Multilevel item response models: An approach to errors in variables regression. Journal of Educational and Behavioral Statistics.

[CR2] Ames AJ, Samonte K (2015). Using SAS PROC MCMC for item response theory models. Educational and Psychological Measurement.

[CR3] Andrich D (1982). An index of person separation in latent trait theory, the traditional KR-20 index, and the Guttman scale response pattern. Educational Research and Perspectives.

[CR4] Asparouhov, T., & Muthén, B. (2010). *Bayesian analysis using Mplus: Technical implementation* [Mplus Technical Report] Retrieved September 17, 2021, from http://statmodel.com/download/Bayes3.pdf. Accessed 17 Sept 2021.

[CR5] Carpenter, B., Gelman, A., Hoffman, M. D., Lee, D., Goodrich, B., Betancourt, M., Brubaker, M., Guo, J., Li, P., & Riddell, A. (2017). Stan: A probabilistic programming language. *Journal of Statistical Software*, *76*(1), 1 - 32. 10.18637/jss.v076.i01 10.18637/jss.v076.i01 PMC9788645 36568334

[CR6] Chalmers, R. P. (2012). mirt: A multidimensional item response theory package for the R environment. *Journal of Statistical Software*, *48*(6), 1-29. 10.18637/jss.v048.i06

[CR7] Choi, Y.-J., & Asilkalkan, A. (2019). R packages for item response theory analysis: Descriptions and features. *Measurement: Interdisciplinary Research and Perspectives*, *17*(3), 168-175. 10.1080/15366367.2019.1586404

[CR8] Congdon, P. (2006). *Bayesian statistical modelling* (2nd ed.). John Wiley & Sons.

[CR9] Dierendonck, C., de Chambrier, A.-F., Fagnant, A., Luxembourger, C., Tinnes-Vigne, M., & Poncelet, D. (2021). Investigating the dimensionality of early numeracy using the bifactor exploratory structural equation modeling framework. *Frontiers in Psychology*, *12*(2195). 10.3389/fpsyg.2021.680124 10.3389/fpsyg.2021.680124 PMC8258407 34239484

[CR10] DiTrapani J, Rockwood N, Jeon M (2018). IRT in SPSS using the SPIRIT macro. Applied Psychological Measurement.

[CR11] Fujimoto, K. A., & Falk, C. F. (2024). The accuracy of Bayesian model fit indices in selecting among multidimensional item response theory models. *Educational and Psychological Measurement*,* 0*(0). 10.1177/00131644231165520 10.1177/00131644231165520 PMC11185105 38898878

[CR12] Fujimoto KA, Neugebauer SR (2020). A general Bayesian multidimensional item response theory model for small and large samples. Educational and Psychological Measurement.

[CR13] Garnier-Villarreal M, Merkle EC, Magnus BE (2021). Between-item multidimensional IRT: How far can the estimation methods go?. Psych.

[CR14] Geisser S, Eddy WF (1979). A predictive approach to model selection. Journal of the American Statistical Association.

[CR15] Gelman A, Rubin DB (1992). Inference from iterative simulation using multiple sequences. Statistical Science.

[CR16] Geyer CJ (1992). Practical Markov chain Monte Carlo. Statistical Science.

[CR17] Gilholm P, Mengersen K, Thompson H (2021). Bayesian hierarchical multidimensional item response modeling of small sample, sparse data for personalized developmental surveillance. Educational and Psychological Measurement.

[CR18] Glockner-Rist A, Hoijtink H (2003). The best of both worlds: Factor analysis of dichotomous data using item response theory and structural equation modeling. Structural Equation Modeling: A Multidisciplinary Journal.

[CR19] Goodman LA (1960). On the exact variance of products. Journal of the American Statistical Association.

[CR20] Hecht M, Gische C, Vogel D, Zitzmann S (2020). Integrating out nuisance parameters for computationally more efficient Bayesian estimation – An illustration and tutorial. Structural Equation Modeling: A Multidisciplinary Journal.

[CR21] Hecht M, Weirich S, Zitzmann S (2021). Comparing the MCMC efficiency of JAGS and Stan for the multi-level intercept-only model in the covariance- and mean-based and classic parametrization. Psych.

[CR22] Hecht M, Zitzmann S (2020). A computationally more efficient Bayesian approach for estimating continuous-time models. Structural Equation Modeling: A Multidisciplinary Journal.

[CR23] IBM Corp. (2019). *IBM SPSS Statistics for Windows, Version 26*. IBM Corp.

[CR24] Kass RE, Carlin BP, Gelman A, Neal RM (1998). Markov Chain Monte Carlo in practice: A roundtable discussion. The American Statistician.

[CR25] König C, Spoden C, Frey A (2020). An optimized Bayesian hierarchical two-parameter logistic model for small-sample item calibration. Applied Psychological Measurement.

[CR26] König C, Spoden C, Frey A (2022). Robustness of the performance of the optimized hierarchical two-parameter logistic IRT model for small-sample item calibration. Behavior Research Methods.

[CR27] Link, W. A., & Eaton, M. J. (2012). On thinning of chains in MCMC. *Methods in Ecology and Evolution*,* 3*(1), 112-115. 10.1111/j.2041-210X.2011.00131.x

[CR28] Liu Y, Yang JS (2018). Interval estimation of latent variable scores in item response theory. Journal of Educational and Behavioral Statistics.

[CR29] Martin, M. O., von Davier, M., & Mullis, I. V. S. (Eds.). (2020). *Methods and Procedures: TIMSS 2019 Technical Report*. TIMSS & PIRLS International Study Center, Lynch School of Education and Human Development, Boston College and International Association for the Evaluation of Educational Achievement (IEA).

[CR30] Merkle, E. C., Fitzsimmons, E., Uanhoro, J., & Goodrich, B. (2021). Efficient Bayesian structural equation modeling in Stan. *Journal of Statistical Software*, *100*(6), 1 - 22. 10.18637/jss.v100.i06

[CR31] Mislevy RJ, Beaton AE, Kaplan B, Sheehan KM (1992). Estimating population characteristics from sparse matrix samples of item responses. Journal of Educational Measurement.

[CR32] Monroe S (2021). Testing latent variable distribution fit in IRT using posterior residuals. Journal of Educational and Behavioral Statistics.

[CR33] Muthén BO (2002). Beyond SEM: General latent variable modeling. Behaviormetrika.

[CR34] Organization for Economic Co-Operation and Development. (2016). *PISA 2015 technical report*. OECD. Retrieved September 3, 2021, from https://www.oecd.org/pisa/data/2015-technical-report/. Accessed 3 Sept 2021.

[CR35] Pohl S, Gräfe L, Rose N (2014). Dealing with omitted and not-reached items in competence tests: Evaluating approaches accounting for missing responses in item response theory models. Educational and Psychological Measurement.

[CR36] Reckase MD (2009). Multidimensional item response theory.

[CR37] Robitzsch A (2021). On the treatment of missing item responses in educational large-scale assessment data: An illustrative simulation study and a case study using PISA 2018 mathematics data. European Journal of Investigation in Health, Psychology and Education.

[CR38] Robitzsch A, Kiefer T, Wu M (2024). *TAM: Test Analysis Modules. R package version 4.2-21*. https://CRAN.Rproject.org/package=TAM. Accessed 14 Mar 2024.

[CR39] Rose, N., von Davier, M., & Nagengast, B. (2016). Modeling omitted and not-reached items in IRT models. *Psychometrika*, 1-25. 10.1007/s11336-016-9544-7 10.1007/s11336-016-9544-7 27848151

[CR40] Rubin DB (1976). Inference and missing data. Biometrika.

[CR41] SAS Institute Inc. (2018). *SAS/STAT® 15.1 user's guide*. SAS Institute Inc.

[CR42] Sheng Y (2013). An empirical investigation of Bayesian hierarchical modeling with unidimensional IRT models. Behaviormetrika.

[CR43] Smid SC, McNeish D, Miočević M, van de Schoot R (2020). Bayesian versus frequentist estimation for structural equation models in small sample contexts: A systematic review. Structural Equation Modeling: A Multidisciplinary Journal.

[CR44] Spiegelhalter, D. J., Best, N. G., Carlin, B. P., & Van Der Linde, A. (2002). Bayesian measures of model complexity and fit. *Journal of the Royal Statistical Society: Series B (Statistical Methodology)*, *64*(4), 583-639. 10.1111/1467-9868.00353

[CR45] Stone, C. A., & Zhu, X. (2015). *Bayesian analysis of item response theory models using SAS*. SAS Institute Inc.

[CR46] van de Schoot R, Winter SD, Ryan O, Zondervan-Zwijnenburg M, Depaoli S (2017). A systematic review of Bayesian articles in psychology: The last 25 years. Psychological Methods.

[CR47] Vehtari, A., Simpson, D., Gelman, A., Yao, Y., & Gabry, J. (2022). Pareto smoothed importance sampling. *arXiv preprint *arXiv:1507.02646v8. 10.48550/arXiv.1507.02646

[CR48] Wagner W, Hecht M, Zitzmann S (2023). A SAS macro for automated stopping of Markov chain Monte Carlo estimation in Bayesian modeling with PROC MCMC. Psych.

[CR49] Watanabe S (2013). A widely applicable Bayesian information criterion. Journal of Machine Learning Research.

[CR50] Wu, M. L., Adams, R. J., Wilson, M. R., & Haldane, S. A. (2007). *ACER ConQuest version 2.0: Generalized item response modelling software*. ACER Press.

[CR51] Zhang, Z., & Zhao, M. (2019). Standard errors of IRT parameter scale transformation coefficients: comparison of bootstrap method, delta method, and multiple imputation method. *Journal of Educational Measurement*, *56*(2), 302-330. 10.1111/jedm.12210

[CR52] Zitzmann S, Hecht M (2019). Going beyond convergence in Bayesian estimation: Why precision matters too and how to assess it. Structural Equation Modeling.

[CR53] Zitzmann S, Lüdtke O, Robitzsch A, Hecht M (2021). On the performance of Bayesian approaches in small samples: A comment on Smid, McNeish, Miocevic, and van de Schoot (2020). Structural Equation Modeling: A Multidisciplinary Journal.

[CR54] Zitzmann S, Weirich S, Hecht M (2021). Using the effective sample size as the stopping criterion in Markov chain Monte Carlo with the Bayes module in Mplus. Psych.

